# Diagnostic delay in early-onset Alzheimer’s disease in Japan: a retrospective study

**DOI:** 10.1186/s12883-026-05121-y

**Published:** 2026-07-09

**Authors:** Kensaku Kasuga, Soichiro Shimizu, Noriyuki Kimura, Koji Kasanuki, Shu Sakurai, Shuntaro Serisawa, Teruaki Masuda, Takuya Ataka, Masanori Tadokoro, Chiaki Hashimoto, Hiroko Shiina, Yosuke Nakamura

**Affiliations:** 1 Department of Molecular Genetics, Brain Research Institute, Niigata University, 1-757 Asahimachidori, Chuo-ku, Niigata, 951-8585 Japan; 2 Department of Diagnostic Innovation Science, Center for Development of Advanced Medicine for Dementia, National Center for Geriatrics and Gerontology, 7-430 Morioka-cho, Obu, Aichi, 474-8511 Japan; 3 Department of Geriatric Medicine, Tokyo Medical University, 6-7-1 Nishishinjuku, Shinjuku-ku, Tokyo 160-0023 Japan; 4 Department of Neurology, Faculty of Medicine, Oita University, 1‑1 Idaigaoka, Hasama-machi, Yufu, Oita, 879‑5593 Japan; 5 Department of Neuropsychiatry, St. Marianna University School of Medicine, 2-16-1 Sugao, Miyamae-ku, Kawasaki, 216-8511 Japan; 6 Alzheimer’s Disease Global Medical Affairs, Eisai Co., Ltd., 4-6-10 Koishikawa, Bunkyo-ku, Tokyo, 112-8088 Japan; 7 Neurology Global Medical Affairs, Eisai Inc., 200 Metro Blvd, Nutley, NJ 07110 USA

**Keywords:** Alzheimer’s disease, Alzheimer’s disease, early onset, Alzheimer’s disease, late onset, Diagnosis delay

## Abstract

**Background:**

Diagnosing early-onset Alzheimer’s disease (EOAD) has unique challenges compared with late-onset AD (LOAD), but the diagnostic journey in Japan is unknown. The objectives of this study were to evaluate the time from symptom onset to diagnosis of EOAD compared with LOAD in Japan, and to investigate factors affecting the time to diagnosis.

**Methods:**

Patients aged between 18 and 79 years who were diagnosed between April 2011 and March 2023 at participating facilities across Japan with “probable AD dementia”, “mild cognitive impairment due to AD”, or an equivalent diagnosis were included. Patients were categorized by age of onset as EOAD (< 65 years) or LOAD (≥ 65 years). Eligible EOAD and time-matched LOAD patients were enrolled. This retrospective observational study was registered in the UMIN Clinical Trials Registry on 28 November 2023 (UMIN000052928).

**Results:**

The mean (standard deviation) time from symptom onset to diagnosis (primary endpoint) was 138.3 (91.0) weeks for EOAD (*n* = 79) and 99.6 (81.5) weeks for LOAD (*n* = 59) (*p* = 0.011). Time from symptom onset to first medical visit was 89.0 (74.2) weeks (EOAD) and 57.6 (71.5) weeks (LOAD) (*p* = 0.024). No difference between EOAD and LOAD was found in the time after the first medical visit to diagnosis.

**Conclusions:**

In Japan, patients with EOAD experienced a longer time to diagnosis than patients with LOAD. Symptoms, regional differences, and access to medical care may have influenced the time to diagnosis. Therefore, it is important to raise awareness of the disease and the need for early consultation, even among non-elderly individuals.

**Trial registration:**

UMIN000052928.

**Supplementary Information:**

The online version contains supplementary material available at https://doi.org/10.1186/s12883-026-05121-y.

## Background

Alzheimer’s disease (AD) is the most common form of dementia, affecting cognition, behavior, functional abilities, and social interactions [1]. The incidence of AD and other dementias increased between 1990 and 2019 [2], though these changes were not uniform worldwide [3]. It is estimated that by 2050 there will be approximately 152 million people with dementia, including patients with AD [4].

AD can be categorized as early-onset AD (EOAD) and late-onset AD (LOAD) by the age of the patient at symptom onset, with EOAD occurring in patients aged < 65 years and LOAD occurring in patients aged ≥ 65 years [5, 6]. In Japan, LOAD accounts for approximately two-thirds (5 million cases) of all forms of dementia, while EOAD accounts for about 50% of early-onset dementia (approximately 35,000 cases) [7]. Patients with EOAD have a higher employment rate than those with LOAD (62% vs. 56%, respectively) [8], resulting in a more severe financial impact of AD (including a decrease in income) for patients under the age of 65 years [9, 10]. Specifically, the work productivity loss due to AD in people aged 50–54 years is more than twice that in those aged 65–69 years [9]. Furthermore, anti-amyloid β (Aβ) antibodies were recently approved in Japan for the treatment of early AD (mild cognitive impairment or mild dementia due to AD) [11, 12]. Therefore, prompt diagnosis of AD is crucial to enable treatment at an early stage to slow disease progression and prevent further loss of productivity [10].

EOAD presents unique diagnostic challenges compared with LOAD, including less pronounced cognitive impairments with a greater occurrence of atypical non-amnestic symptoms (e.g., aphasia, apraxia) and differences in neuroimaging patterns [5, 13–16]. These factors, combined with the younger age at onset, can lead to misdiagnosis or delayed diagnosis [5, 13–15, 17], and there is a need for greater clinical awareness and comprehensive evaluation in younger patients with variable symptoms. However, there have been few studies that have investigated the time to AD diagnosis, and no such studies in Asian countries, including Japan, have been conducted.

Therefore, this study was conducted to evaluate the diagnostic journey from the time of symptom onset to diagnosis of EOAD compared with LOAD in Japan, and to investigate factors that may influence the time to diagnosis.

## Methods

### Study design

**Fig. 1:**
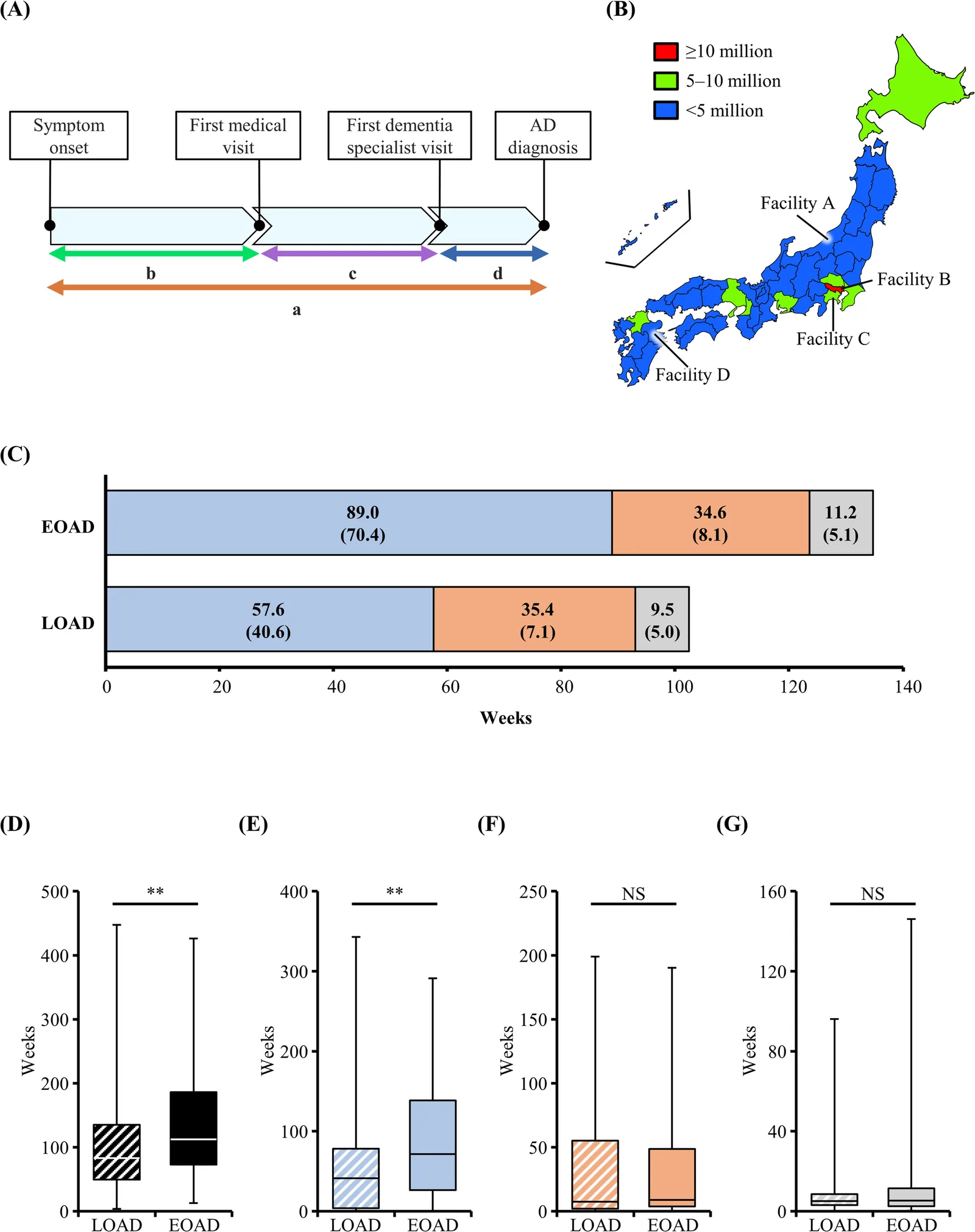
Geographic location of each facility and time from symptom onset to AD diagnosis. **(A)** Study design. a (orange line) refers to the time from symptom onset to AD diagnosis, b (green line) refers to time from symptom onset to the first medical visit, c (purple line) refers to time from the first medical visit to the first dementia specialist visit, and d (blue line) refers to time from the first dementia specialist visit to AD diagnosis. Information was determined from retrospective extraction and analysis of electronic medical records. **(B)** Four facilities participated in the study across Japan. The prefectures in blue are those with fewer than 5 million people, the prefectures in green have 5 to 10 million people, and the prefecture in red has greater than 10 million people. **(C)** Mean (median) weeks from symptom onset to the first medical visit, the first dementia specialist visit, and AD diagnosis. The blue portion refers to the time from symptom onset to the first medical visit; orange refers to time from the first medical visit to the first dementia specialist visit; and gray refers to time from the first dementia specialist visit to AD diagnosis. **(D)**–**(G)** Box-and-whisker plots showing median time from symptom onset to AD diagnosis **(D)**, median time from symptom onset to the first medical visit **(E)**, median time from the first medical visit to the first dementia specialist visit **(F)**, and median time from the first dementia specialist visit to AD diagnosis **(G)**. **p ≤ 0.01 (Wilcoxon rank sum test). AD: Alzheimer’s disease; EOAD: early-onset Alzheimer’s disease; LOAD: late-onset Alzheimer’s disease; NS: not significant

This multicenter, retrospective observational study examined the diagnostic journey of EOAD and LOAD in Japan (Fig. 1A). The study was performed in accordance with the ethical standards of the 1964 Declaration of Helsinki and its later amendments of comparable ethical standards. The Ethics Committee of Niigata University approved the research plan. The study was registered in the University hospital Medical Information Network Center database under the identifier UMIN000052928.

### Patients

Eligible patients were those with a diagnosis of “probable AD dementia [18],” “mild cognitive impairment due to AD [19],” or equivalent, and diagnosed at the participating facilities during the eligibility period (from April 2011 to March 2023), regardless of the confirmation of the presence of Aβ by imaging or cerebrospinal fluid (CSF) tests. Therefore, all patients in this study were diagnosed by a dementia specialist. Each patient was classified as EOAD (< 65 years) or LOAD (≥ 65 years) based on their age at symptom onset. Symptom onset was defined as the time when the first cognitive and/or behavioral symptoms were recognized by the patient, their family members, or other persons, as described in the electronic medical record.

Data from four facilities in Japan were included: Facility A, which serves a rural community of fewer than 5 million people; Facility B, which serves an urban community of greater than 10 million people; Facility C, which serves an urban community of 5 to 10 million people; and Facility D, which serves a rural community of fewer than 5 million people (Fig. 1B).

All EOAD patients who were diagnosed during the eligibility period and did not opt out of the study were included. Eligible EOAD patients and time-matched LOAD patients were enrolled. Other details are described in the Supplemental Methods.

### Endpoints

The primary endpoint was the time from symptom onset to diagnosis of AD. The secondary endpoint was the factors affecting the time from symptom onset to AD diagnosis. The types of data collected are listed in the Supplemental Methods.

All cognitive and psychological scales used in this study including Alzheimer’s Disease Assessment Scale-Cognitive Subscale (ADAS-cog) [20], Clinical Dementia Rating (CDR) [21], Frontal Assessment Battery (FAB) [22], Geriatric Depression Scale (GDS) [23], Hasegawa Dementia Scale-revised (HDS-R) [24], Montreal Cognitive Assessment-Japanese version (MoCA-J) [25], Mini-Mental State Examination (MMSE) [26], Self-rating Depression Scale (SDS) [27], St. Marianna University School of Medicine, Computerized Memory Test (STM-COMET) [28, 29], Trail Making Test (TMT) [30, 31], and Wechsler Memory Scale-revised, logical memory (WMS-R, LM) [32] have been previously validated.

### Statistical analysis

Between-group comparisons were conducted using Student’s *t*-test to compare means, Fisher’s exact test was used to compare categorical data, and Wilcoxon’s rank sum test was used to compare medians.

Multiple regression analysis was conducted for the secondary endpoint using the time from symptom onset to the diagnosis of AD (weeks) as the objective variable. Explanatory variables were identified using a stepwise regression method and selected based on their clinical relevance. These included primary medical care provider-related and dementia specialist-related variables (Supplemental Table 1).

The significance level was set at 5% (two-sided). Missing data were not imputed. All statistical analyses were performed using SAS Release 9.4 TS1M4 (×64 version or later) (SAS Institute, Cary NC, USA).

## Results

### Patients

A total of 146 patients were enrolled and met the eligibility criteria, including 83 patients with EOAD and 63 time-matched patients with LOAD. Eight patients (EOAD *n* = 4, LOAD *n* = 4) were excluded by the decision of the physician because AD was diagnosed during an outpatient visit owing to a comorbidity (EOAD *n* = 1) or because the patient was diagnosed without the requisite referral from a clinic or another institution (EOAD *n* = 3, LOAD *n* = 4). Data for 138 patients were analyzed (79 patients with EOAD and 59 patients with LOAD).

The mean age at symptom onset was 72.0 years in patients with LOAD and 55.8 years in patients with EOAD (Table 1). The background characteristics of both groups were comparable, except for “presence of established medical care provider”, “department of the first medical care provider that the patient visited”, “presence of on-treatment comorbidities at symptom onset”, “educational level”, and “employment status”.

**Table 1:** Patient background characteristics

Background of patients		LOAD (*n* = 59)	EOAD (*n* = 79)	
Facility	A	32 (54.2)	48 (60.8)	*p* = 0.850^*^
	B	10 (16.9)	10 (12.7)	
	C	7 (11.9)	8 (10.1)	
	D	10 (16.9)	13 (16.5)	
Sex	Male	20 (33.9)	33 (41.8)	*p* = 0.380^*^
	Female	39 (66.1)	46 (58.2)	
Presence of established medical care provider	Yes	52 (88.1)	43 (54.4)	*p* < 0.001^*^
	No	4 (6.8)	23 (29.1)	
	Unknown	3 (5.1)	13 (16.5)	
Individual who initially noticed symptom onset	Patient	12 (20.3)	21 (26.6)	
	Family member	44 (74.6)	49 (62.0)	
	Friend	3 (5.1)	2 (2.5)	
	Colleague in the workplace	1 (1.7)	20 (25.3)	
	Medical worker	4 (6.8)	2 (2.5)	
	Other	2 (3.4)	5 (6.3)	
	Unknown	5 (8.5)	7 (8.9)	
Department of the first medical care provider that the patient visited	Neurology	9 (15.3)	27 (34.2)	*p* < 0.001^*^
	Internal medicine	29 (49.2)	15 (19.0)	
	Psychiatry	2 (3.4)	8 (10.1)	
	Gerontology	0 (0.0)	0 (0.0)	
	Neurosurgery	6 (10.2)	19 (24.1)	
	Others	13 (22.0)	4 (5.1)	
	Unknown	0 (0.0)	6 (7.6)	
Age at first medical care provider visit	Mean ± SD (y)	72.7 ± 3.9	57.6 ± 5.3	*t* = − 16.71^†^ *p* < 0.001^†^ Z = 9.18^‡^ *p* < 0.001^‡^
	Median (y)	72.0	58.0	
	Unknown (n)	13	5	
Marital status at symptom onset	Married	47 (79.7)	65 (82.3)	*p* = 0.587^*^
	No spouse	5 (8.5)	10 (12.7)	
	Unknown	7 (11.9)	4 (5.1)	
Household status at symptom onset	Living together	50 (84.7)	65 (82.3)	*p* = 0.790^*^
	Living alone	6 (10.2)	10 (12.7)	
	Unknown	3 (5.1)	4 (5.1)	
Employment status at symptom onset	Employed	14 (23.7)	52 (65.8)	*p* < 0.001^*^
	Unemployed	28 (47.5)	17 (21.5)	
	Unknown	17 (28.8)	10 (12.7)	
Presence of on-treatment comorbidities at symptom onset	Present	50 (84.7)	36 (45.6)	*p* < 0.001^*^
	Absent	5 (8.5)	27 (34.2)	
	Unknown	4 (6.8)	16 (20.3)	
Number of on-treatment comorbidities at symptom onset	0	5 (8.5)	27 (34.2)	*p* < 0.001^*^ Z = 4.87^‡^ *p* < 0.001^‡^
	1–2	25 (42.4)	29 (36.7)	
	3–4	23 (39.0)	5 (6.3)	
	5–6	1 (1.7)	2 (2.5)	
	7–8	1 (1.7)	0 (0.0)	
	≥ 9	0 (0.0)	0 (0.0)	
Family history of AD (limited to biological parents, grandparents, and siblings)	Present	11 (18.6)	26 (32.9)	*p* = 0.363^*^
	Absent	18 (30.5)	27 (34.2)	
	Unknown	30 (50.8)	26 (32.9)	
Age at symptom onset	Mean ± SD, y	72.0 ± 3.8	55.8 ± 5.4	*t* = − 19.63^†^ *p* < 0.001^†^ Z = 10.04^‡^ *p* < 0.001^‡^
	Median, y	72.0	56.0	
Educational level	< 9 y	2 (3.4)	0 (0.0)	*p* < 0.001^*^ Z = − 2.41^‡^ *p* = 0.016^‡^
	9–<12 y	8 (13.6)	4 (5.1)	
	12–<14 y	29 (49.2)	35 (44.3)	
	14–<16 y	0 (0.0)	16 (20.3)	
	≥ 16 y	10 (16.9)	12 (15.2)	
	Unknown	10 (16.9)	12 (15.2)	
Number of visits to medical care providers from symptom onset to diagnosis	< 3	2 (3.4)	2 (2.5)	*p* = 0.071^*^ Z = − 0.96^‡^ *p* = 0.339^‡^
	3–4	26 (44.1)	23 (29.1)	
	5–6	10 (16.9)	30 (38.0)	
	7–9	10 (16.9)	10 (12.7)	
	≥ 10	4 (6.8)	4 (5.1)	
	Unknown	7 (11.9)	10 (12.7)	
Initial symptoms* (symptoms at 3 years after onset of AD)	Cognitive impairment	58 (98.3)	74 (93.7)	
	Aphasia (loss of speech)	5 (8.5)	12 (15.2)	
	Visuospatial agnosia	6 (10.2)	12 (15.2)	
	Executive dysfunction	11 (18.6)	34 (43.0)	
	Anxiety and depression	4 (6.8)	10 (12.7)	
	Personality changes	7 (11.9)	7 (8.9)	
	Loss of control over behavior	3 (5.1)	15 (19.0)	
	Other	7 (11.9)	12 (15.2)	
Neuropsychological test performed	MMSE	57 (96.6)	66 (83.5)	
	HDS-R	24 (40.7)	26 (32.9)	
	WMS-R, LM	23 (39.0)	26 (32.9)	
	CDR	16 (27.1)	29 (36.7)	
	MoCA-J	17 (28.8)	19 (24.1)	
	ADAS-cog	16 (27.1)	20 (25.3)	
	TMT	8 (13.6)	18 (22.8)	
	GDS	12 (20.3)	12 (15.2)	
	FAB	4 (6.8)	9 (11.4)	
	SDS	3 (5.1)	6 (7.6)	
	STM-COMET	6 (10.2)	2 (2.5)	
	Other	16 (27.1)	21 (26.6)	

Data are *n* (%) unless otherwise indicated

*AD* Alzheimer’s disease, *ADAS-cog* Alzheimer’s Disease Assessment Scale-Cognitive Subscale, *CDR* Clinical Dementia Rating, *EOAD* early-onset Alzheimer’s disease, *FAB* Frontal Assessment Battery, *GDS* Geriatric Depression Scale, *HDS-R* Hasegawa Dementia Scale-revised, *LOAD* late-onset Alzheimer’s disease, *MoCA-J* Montreal Cognitive Assessment-Japanese version, *MMSE* Mini-Mental State Examination, *SD* standard deviation, *SDS* Self-rating Depression Scale, *STM-COMET* St Marianna University School of Medicine’s Computerized Memory Test, *TMT* Trail Making Test, *WMS-R*, *LM* Wechsler Memory Scale-revised, logical memory

^*^Fisher’s exact test

^†^Student’s *t*-test

^‡^Wilcoxon rank sum test

^§^Symptoms that were present during the first 3 years after AD onset

Cognitive impairment was the most common and the most prominent initial symptom in both patients with EOAD and LOAD (Supplemental Fig. 1). Patients with EOAD were more likely to have aphasia, visuospatial agnosia, executive dysfunction, anxiety/depression, and apraxia as initial symptoms than patients with LOAD. Magnetic resonance imaging (MRI) was less likely to be performed in patients with EOAD (EOAD: 38.0%, LOAD: 71.2%; *p* < 0.001) in the dementia specialist facilities because it had already been conducted by the medical provider who referred the patient to the dementia specialist (Supplemental Fig. 2A). Amyloid positron emission tomography (PET) scans were less likely to be performed in patients with LOAD (EOAD: 16.5%, LOAD: 3.4%; *p* = 0.024). Patients with EOAD were more likely to have had CSF tests (EOAD: 36.7%, LOAD: 13.6%; *p* = 0.003) and less likely to have had a Mini-Mental State Examination than those with LOAD (EOAD: 83.5%, LOAD: 96.6%; *p* = 0.024) (Supplemental Fig. 2B and 2 C).

### Primary endpoint

Differences in the time from symptom onset to AD diagnosis between patients with EOAD and LOAD are shown in Fig. 1C and Supplemental Fig. 3. The mean ± standard deviation time from symptom onset to diagnosis was 138.3 ± 91.0 weeks for patients with EOAD and 99.6 ± 81.5 weeks for patients with LOAD (*p* = 0.011). The time from symptom onset to the first medical visit was 89.0 ± 74.2 weeks for patients with EOAD and 57.6 ± 71.5 weeks for patients with LOAD (*p* = 0.024). There was no between-group difference in the time from the first medical visit to the first dementia specialist visit and the first dementia specialist visit to AD diagnosis (*p* = 0.932 and 0.596, respectively).

Similar delay for patients with EOAD was found in the sensitivity analysis that excluded outliers (data exceeding 3 standard deviations from the mean) (Supplemental Table 2).

### Secondary endpoints

#### Factors affecting the time from symptom onset to AD diagnosis

In the overall population (combined population of EOAD and LOAD), multiple regression analysis found that relative to the neurology department as the “department of the first medical care provider that the patient visited,” internal medicine or neurosurgery departments were associated with a shorter time from symptom onset to diagnosis (internal medicine: β = −57.25, *p* = 0.024; neurosurgery: β = −58.81, *p* = 0.008) (Table 2). Consistent results with the primary endpoint, diagnosis of EOAD was associated with a longer time from symptom onset to diagnosis relative to LOAD diagnosis (β = 34.33, *p* = 0.060).

**Table 2:** Factors affecting the time from symptom onset to AD diagnosis

Factors		Overall population				LOAD				EOAD			
		Estimate	SE	t value	*p* value	Estimate	SE	t value	*p* value	Estimate	SE	t value	*p* value
Intercept		160.41	35.22	4.55	< 0.001	194.42	97.85	1.99	0.054	166.20	35.43	4.69	< 0.001
Diagnosis^*^	EOAD	34.33	18.04	1.90	0.060								
	LOAD	0.00											
Facility^†^	A	0.00				0.00				0.00			
	B	−66.94	43.71	−1.53	0.128	−58.91	69.68	−0.85	0.403	−114.71	60.18	−1.91	0.062
	C	21.16	33.82	0.63	0.533	−31.66	63.30	−0.50	0.620	12.24	49.82	0.25	0.807
	D	−2.45	38.19	−0.06	0.949	4.16	61.34	0.07	0.946	−21.91	67.39	−0.33	0.746
Initial symptoms^‡^ (symptoms at 3 years after onset of AD)	Aphasia	−25.65	23.34	−1.10	0.274	−187.79	56.14	−3.34	0.002	0.50	29.83	0.02	0.987
	Visuospatial agnosia	−36.35	22.29	−1.63	0.106	−95.79	42.34	−2.26	0.030	−13.70	28.93	−0.47	0.638
Department of the first medical care provider that the patient visited^§^	Neurology	0.00				0.00				0.00			
	Internal medicine	−57.25	25.07	−2.28	0.024	−155.85	46.66	−3.34	0.002	−72.86	34.13	−2.13	0.037
	Psychiatry	−52.26	33.01	−1.58	0.116	−143.80	71.97	−2.00	0.053	−56.54	43.58	−1.30	0.200
	Neurosurgery	−58.81	21.59	−2.72	0.008	−150.45	44.27	−3.40	0.002	−53.94	26.39	−2.04	0.046
	Other + Unknown	−9.06	24.04	−0.38	0.707	−154.66	42.15	−3.67	< 0.001	66.55	35.80	1.86	0.068

The objective variable is the time from symptom onset to definitive diagnosis

*AD* Alzheimer’s disease, *EOAD* early-onset Alzheimer’s disease, *LOAD* late-onset Alzheimer’s disease, *SE* standard error

*Reference: LOAD

^†^Reference: Facility A

^‡^Two values (applicable and not applicable)

^§^Reference: Neurology

When assessed by the type of diagnosis (EOAD or LOAD), relative to neurology department as the “department of the first medical care provider that the patient visited,” internal medicine and neurosurgery departments were associated with a shorter time to diagnosis in both groups (EOAD, internal medicine: β = −72.86, *p* = 0.037; neurosurgery: β = −53.94, *p* = 0.046; LOAD, internal medicine: β = −155.85, *p* = 0.002; neurosurgery: β = −150.45, *p* = 0.002). In the EOAD group, registration to Facility B showed a trend towards a shorter time to diagnosis relative to Facility A (β = −114.71, *p* = 0.062). In the LOAD group, the initial symptoms of aphasia or visuospatial agnosia were both associated with a shorter time from symptom onset to diagnosis (aphasia: β = −187.79, *p* = 0.002; visuospatial agnosia: β = −95.79, *p* = 0.030). 

#### Factors affecting the time from symptom onset to the first medical visit

In the overall population, diagnosis of EOAD was associated with a longer time from symptom onset to the first medical visit relative to a diagnosis of LOAD (β = 42.50, *p* = 0.011) (Table 3), consistent with the results shown in Fig. 1C. Relative to registration to Facility A, registration to Facility B (β = −103.09, *p* = 0.020) was associated with a shorter time to the first medical visit. Relative to neurology as the “department of the first medical care provider that the patient visited,” neurosurgery was also associated with a shorter time to the first medical visit (β = −42.31, *p* = 0.028).

**Table 3:** Factors affecting the time from symptom onset to the first medical visit

Factors		Overall population				LOAD				EOAD			
		Estimate	SE	t value	*p* value	Estimate	SE	t value	*p* value	Estimate	SE	t value	*p* value
Intercept		96.81	30.99	3.12	0.002	90.31	107.55	0.84	0.409	138.12	32.07	4.31	< 0.001
Diagnosis^*^	EOAD	42.50	16.30	2.61	0.011								
	LOAD	0.00											
Facility^†^	A	0.00				0.00				0.00			
	B	−103.09	43.47	−2.37	0.020	−3.42	77.24	−0.04	0.965	−194.69	68.18	−2.86	0.006
	C	−17.17	29.36	−0.58	0.560	−5.16	67.96	−0.08	0.940	−33.99	46.54	−0.73	0.468
	D	0.59	39.67	0.01	0.988	25.46	89.34	0.28	0.778	−56.37	62.08	−0.91	0.368
Department of the first medical care provider that the patient visited^‡^	Neurology	0.00				0.00				0.00			
	Internal medicine	−42.02	22.70	−1.85	0.067	−113.80	47.61	−2.39	0.025	−34.19	32.33	−1.06	0.295
	Psychiatry	−36.70	28.70	−1.28	0.204	−116.01	94.81	−1.22	0.233	−28.65	39.47	−0.73	0.471
	Neurosurgery	−42.31	18.97	−2.23	0.028	−91.57	44.98	−2.04	0.053	−26.45	23.94	−1.10	0.274
	Other + Unknown	0.60	22.04	0.03	0.978	−30.76	40.10	−0.77	0.450	13.04	35.08	0.37	0.712

The objective variable is the time from symptom onset to first visit

*AD* Alzheimer’s disease, *EOAD* early-onset Alzheimer’s disease, *LOAD* late-onset Alzheimer’s disease, *SE* standard error

^*^Reference: LOAD

^†^Reference: Facility A

^‡^Reference: Neurology

In the EOAD group, relative to registration to Facility A, Facility B was associated with a shorter time from symptom onset to the first medical visit (β = −194.69, *p* = 0.006). In the LOAD group, relative to neurology as the “department of the first medical care provider that the patient visited,” internal medicine was associated with a shorter time to the first medical visit (β = −113.80, *p* = 0.025). 

#### Factors affecting the time from the first medical visit to the first dementia specialist visit

No intergroup difference in the time from the first medical visit to the first dementia specialist visit was observed (Fig. 1C). In the overall population, the patient as the “individual who initially noticed symptom onset” (β = 37.09, *p* < 0.001), cognitive impairment as the “initial symptom” (β = 55.05, *p* = 0.046), and living together as the “household status at symptom onset” (β = 33.76, *p* = 0.007) were associated with a longer time from the first medical visit to the first dementia specialist visit (Table 4). Higher “educational level” was associated with a shorter time (β = −5.81, *p* = 0.009).

**Table 4:** Factors affecting the time from the first medical visit to the first dementia specialist visit

Factors		Overall population				LOAD				EOAD			
		Estimate	SE	t value	*p* value	Estimate	SE	t value	*p* value	Estimate	SE	t value	*p* value
Intercept		11.87	48.43	0.25	0.807	62.11	54.03	1.15	0.265	−17.11	64.76	−0.26	0.793
Diagnosis*	EOAD	21.23	10.80	1.97	0.053								
	LOAD	0.00											
Individual who initially noticed symptom onset^†^	Patient	37.09	10.66	3.48	< 0.001	52.26	18.05	2.90	0.009	25.05	16.16	1.55	0.128
Initial symptoms^†^	Cognitive impairment	55.05	27.15	2.03	0.046					56.11	29.02	1.93	0.060
	Other	−25.36	12.51	−2.03	0.046	−12.89	18.95	−0.68	0.505	−28.67	17.00	−1.69	0.099
Household status at symptom onset^‡^	Living together	33.76	12.18	2.77	0.007	−84.12	33.27	−2.53	0.021	48.45	15.75	3.08	0.004
	Living alone + unknown	0.00				0.00				0.00			
Educational level^§^		−5.81	2.17	−2.68	0.009	−2.81	2.54	−1.11	0.283	−4.46	3.32	−1.34	0.187
Imaging^†^	MRI	4.12	11.68	0.35	0.725	81.09	29.64	2.74	0.013	0.41	15.51	0.03	0.979
Neuropsychological tests^†^	WMS-R, LM	11.01	10.65	1.03	0.305	−14.09	15.10	−0.93	0.363	29.09	14.16	2.05	0.046

The objective variable is the time from initial visit to the first dementia specialist visit

*AD* Alzheimer’s disease, *EOAD* early-onset Alzheimer’s disease, *LOAD* late-onset Alzheimer’s disease, *MRI* magnetic resonance imaging, *SE* standard error, *WMS-R, LM* Wechsler Memory Scale-revised, logical memory

*Reference: LOAD

^†^Two values (applicable and not applicable)

^‡^Reference: Living alone + unknown

^§^Continuous variable

In the EOAD group, living together as the “household status at symptom onset” (β = 48.45, *p* = 0.004) and having a Wechsler Memory Scale-revised, logical memory (WMS-R, LM) test (a test performed by the dementia specialist) as a “neuropsychological test” (β = 29.09, *p* = 0.046) were both associated with a longer time from the first medical visit to the first dementia specialist visit. In the LOAD group, the patient as the “individual who initially noticed symptom onset” (β = 52.26, *p* = 0.009) and MRI as an “imaging test” (β = 81.09, *p* = 0.013) were associated with a longer time from the first medical visit to the first dementia specialist visit. Living together as the “household status at symptom onset” (β = −84.12, *p* = 0.021) was associated with a shorter time from first medical visit to the first dementia specialist visit in the LOAD group. 

#### Factors affecting the time from the first dementia specialist visit to AD diagnosis

No intergroup difference in the time from the first dementia specialist visit to AD diagnosis was observed (Fig. 1C). A greater “number of visits to medical care providers from symptom onset to diagnosis” was associated with a longer time from the first dementia specialist visit to AD diagnosis in the overall population, EOAD and LOAD groups (β = 2.71, β = 3.32 and β = 2.71, respectively; all *p* < 0.001) (Table 5).

**Table 5:** Factors affecting the time from the first dementia specialist visit to AD diagnosis

Factors		Overall population				LOAD				EOAD			
		Estimate	SE	t value	*p* value	Estimate	SE	t value	*p* value	Estimate	SE	t value	*p* value
Intercept		−10.04	7.29	−1.38	0.172	−8.40	8.60	−0.98	0.338	−21.18	10.93	−1.94	0.059
Diagnosis*	EOAD	0.56	3.59	0.16	0.876								
	LOAD	0.00											
Number of visits to a medical facility until definitive diagnosis of AD^†^		2.71	0.31	8.86	< 0.001	2.71	0.16	16.78	< 0.001	3.32	0.91	3.64	< 0.001

The objective variable is the time from consultation with a dementia specialist to a definitive diagnosis of AD

*AD* Alzheimer’s disease, *EOAD* early-onset Alzheimer’s disease, *LOAD* late-onset Alzheimer’s disease, *SE* standard error

*Reference: LOAD

^†^Continuous variable

## Discussion

This was the first study to examine the diagnostic journey of patients with EOAD and LOAD in Japan, including factors affecting diagnostic time. Our results demonstrated that patients in Japan with EOAD faced a longer diagnostic time than patients with LOAD, which was primarily attributed to a prolonged time between the symptom onset and the first medical visit. No difference between EOAD and LOAD was found in the time after the first medical visit to diagnosis, which highlights the importance of prompt initial consultations following the onset of symptoms to shorten the diagnostic journey.

Although there were no studies investigating the time to diagnosis of EOAD relative to LOAD in Japan with which to compare our findings, the long diagnostic time experienced by patients with EOAD is consistent with reports from other countries. In Norway, previous studies have reported a mean time from symptom onset to EOAD diagnosis of 5.5 ± 2.8 years [33] and 3.3 ± 1.7 years [34]. In the Netherlands, a study reported that patients with EOAD had a 1.2-year longer delay in diagnosis compared with patients with LOAD (4.2 ± 3.0 years and 3.0 ± 2.2 years, respectively) [35]. Another study found that patients had a mean time from symptom onset to diagnosis of 3.4 ± 2 years (EOAD) and 2.5 ± 2 years (LOAD) [36]. In Australia, patients with EOAD had a median time from symptom onset to diagnosis of approximately 1.5 years [37], and a more recent study reported a mean time from symptom onset to EOAD diagnosis of 2.9 ± 1.7 years [38]. Another study in Sweden estimated that the mean time from onset to diagnosis was 4.1 ± 3.4 years (EOAD) and 2.9 ± 1.7 years (LOAD) [39]. Although the diagnostic criteria varied among the studies, patients with EOAD in our study experienced a similar delay in diagnosis (2.7 ± 1.7 years) relative to LOAD diagnosis (1.9 ± 1.6 years), as reported in other countries. The relatively shorter time to AD diagnosis in Japan as compared to previous studies may be due to differences in the healthcare systems among the countries, as well as the recent increase in the awareness of dementia.

### Factors affecting the time from symptom onset to AD diagnosis

This study showed that the department of the first medical care provider that the patient visited (i.e., internal medicine and neurosurgery) was associated with a shorter time from symptom onset to diagnosis in both EOAD and LOAD groups. Initial symptoms of aphasia and visuospatial agnosia were also associated with a shorter time from symptom onset to diagnosis for patients with LOAD. Aphasia and visuospatial agnosia are atypical symptoms of LOAD, even rarer than in EOAD [36]. However, they may overlap with alarming symptoms reported by patients visiting neurosurgery departments, where prompt medical action may be necessary due to the underlying condition. Such symptoms can also be present in patients with cerebrovascular dementia [40], potentially signaling the need for urgent care. Thus, patients exhibiting these initial symptoms may have been quickly identified by both family members and medical care providers, potentially shortening the time to diagnosis.

### Factors affecting the time from symptom onset to the first medical visit

For patients with EOAD, being registered at Facility B (relative to Facility A) was associated with a shorter time to the first medical visit. Facility B was in an urban setting and served a large community (> 10 million people), whereas Facility A served a rural community (< 5 million people). These regional differences were consistent with previous studies comparing rural and urban differences in the use of health services [41, 42], highlighting the importance of a standardized care pathway. For instance, patients with dementia in rural areas visited family physicians less frequently than those in urban areas [42].

For patients with LOAD, initially visiting an internal medicine department was associated with a shorter time to the first medical visit. In Japan, internal medicine is the most common specialty among primary care physicians [43]. In this study, a higher percentage of patients with LOAD had an established medical care provider (e.g., general practitioner, primary care physician, or specialist) and comorbidities at symptom onset than patients with EOAD. Thus, patients with an established relationship with a medical care provider may have had earlier opportunities for consultation as such providers emphasize patient-centered communication [44]. This may explain the shorter time from symptom onset to the first medical visit. Notably, these findings highlight the importance of considering the age of onset [45], differences in the settings of facilities, and initial point of care when examining factors affecting timely AD diagnosis.

### Factors affecting the time from the first medical visit to the first dementia specialist visit

In this study, household status at symptom onset and having a WMS-R, LM test affected the time from the first medical visit to the first dementia specialist visit for patients with EOAD. The first medical care provider may have quickly referred patients living alone to a dementia specialist out of concern due to their living status. Having the WMS-R, LM test at the specialist relative to its absence was associated with a longer time from the first medical visit to the first dementia specialist visit. Thus, the absence of the WMS-R, LM test was associated with a shorter time to the specialist visit. As this test specifically assesses memory, patients with non-amnestic symptoms (i.e., WMS-R, LM not required) might have a shorter time to the dementia specialist visit because of their complex symptoms. Non-amnestic atypical symptoms such as aphasia, executive dysfunction, anxiety/depression, and apraxia are more likely to appear as initial symptoms in patients with EOAD than in those with LOAD, contributing to a more complex symptom manifestation [5, 14, 15]. Presentation of cognitive impairment with other atypical AD symptoms, as well as the younger age of patients with EOAD, may have contributed to the association between having a WMS-R, LM test and a longer time from the first medical visit to the first dementia specialist visit.

Among patients with LOAD, those who first self-identified symptoms of AD experienced a longer time between the first medical visit and the first dementia specialist visit. This is consistent with a previous study that reported the diagnostic time was longer for patients who visited a primary care physician by themselves, compared with those who were accompanied by a work colleague or family member who had noticed AD symptoms [33]. Thus, the referral to a dementia specialist may be expedited at the request of a colleague or family member.

### Factors affecting the time from the first dementia specialist visit to AD diagnosis

For both EOAD and LOAD, a higher number of pre-diagnosis medical visits was associated with a longer time from the first dementia specialist visit to AD diagnosis. This reflects the longer time required for a greater number of medical visits to occur. For both groups, most neuropsychological tests were performed at the first dementia specialist visit, and most imaging tests were conducted within 8 weeks after the first visit at the specialist (likely corresponding to the second or third specialist visits; data not shown). This suggests that most patients received a prompt diagnosis after visiting the dementia specialist.

The overall rate of amyloid PET use in this study was low (EOAD: 16.5%; LOAD: 3.4%). This likely reflects the limited use of amyloid PET in routine clinical practice during the study period, particularly before the availability of anti-amyloid therapies. Patients with EOAD generally have fewer comorbid neuropathologies than those with LOAD [46, 47], suggesting that patients with EOAD may gain greater benefits from these therapies. However, our study and others found that patients with EOAD experienced long diagnostic delays [35, 36, 39], potentially leading to productivity losses and other financial burdens [10], and may hinder timely access to anti-amyloid therapies. These findings highlight the importance of early diagnosis.

This study had some limitations. As this was a retrospective study based on electronic medical records, only available data could be analyzed, and missing or unrecorded information could not be assessed. There was a potential selection bias, as patients were included on the basis of clinical diagnosis (biomarker assessments were not mandatory), and those diagnosed with AD outside of the participating facilities were excluded. Although the sample size was small, a key strength of this study was its multicenter design, which enabled the inclusion of a relatively large number of patients with EOAD, despite its rarity.

## Conclusions

There is a long period from symptom onset to the first medical visit for patients with EOAD in Japan, contributing to a delay in reaching an AD diagnosis. Our results suggest that the medical environment of the study participants, including regional differences and their level of disease awareness, may have influenced the time taken to obtain an AD diagnosis. With the recent emergence of new treatment options for AD, it is essential to increase the awareness of AD in Japan. Efforts to standardize care pathways, particularly for patients with EOAD, may help to reduce delays in diagnosis and treatment across different healthcare settings. Furthermore, the recent development and anticipated clinical implementation of blood-based biomarkers are expected to facilitate less invasive and more accessible diagnosis, potentially shortening the time from symptom onset to diagnosis. 

## Supplementary Information

Supplementary Material 1.

## Data Availability

The data that support the study findings are included in the article and Supplementary materials. Further inquiries can be directed to the corresponding author.

## Abbreviations

Aβ: Amyloid β
ADAS-cog: Alzheimer’s Disease Assessment Scale-Cognitive Subscale
AD: Alzheimer’s disease
CDR: Clinical Dementia Rating
CSF: Cerebrospinal fluid
EOAD: Early-onset Alzheimer’s disease
FAB: Frontal Assessment Battery
GDS: Geriatric Depression Scale
HDS-R: Hasegawa Dementia Scale-revised
LOAD: Late-onset Alzheimer’s disease
MMSE: Mini-Mental State Examination
MoCA-J: Montreal Cognitive Assessment-Japanese version
MRI: Magnetic resonance imaging
NS: Not significant
PET: Positron emission tomography
SD: Standard deviation
SDS: Self-rating Depression Scale
SE: Standard error
STM-COMET: St Marianna University School of Medicine’s Computerized Memory Test
TMT: Trail Making Test
WMS-R, LM: Wechsler Memory Scale-revised, logical memory
