# Supplementary material for: Diagnostic delay in early-onset Alzheimer’s disease in Japan: a retrospective study
Source: BMC Neurol. 2026 Jul 9;26:620. doi: 10.1186/s12883-026-05121-y (PMC13632443; doi:10.1186/s12883-026-05121-y)
Supplement: Supplementary file 1 [file 12883_2026_5121_MOESM1_ESM.docx]

**Supplemental Material**

**Supplemental Methods**

*Inclusion of patients*

Patients classified as having late-onset Alzheimer’s disease (LOAD) were included according to the following methods to reduce the effect of changes to the medical environment and potential case selection bias.

1. The same number of patients with LOAD who had their first visit to the facility in the same year and month as the registered patients with early-onset AD (EOAD) were enrolled. Patients with LOAD were enrolled in the same order as patients with EOAD, and in order of the earlier date of visit.
2. If there were fewer patients with LOAD than patients with EOAD within the same month and year, patients with LOAD who were examined during the following month or two months were registered in the order of the earlier date of examination.
3. If equal numbers of patients with LOAD and EOAD could not be registered by using the same month plus patients with LOAD who were examined in the following month or the two following months, patients with LOAD who were examined for the first time in the previous month were registered in order of their earlier examination dates.
4. If there remained a shortage of patients with LOAD, those who were examined for the first time in the two previous months were registered.

If the number of patients with LOAD or EOAD were still unequal, even after including patients with LOAD examined during the two months before and after, the number of patients with EOAD and patients with LOAD were treated as unmatched.

*Exclusion of patients*

Patients whose symptom onset or diagnosis could not be confirmed, those with a diagnosis of dementia due to diseases other than AD, or those the investigator deemed inappropriate for study inclusion were excluded.

*Items surveyed*

The dates of symptom onset, the first medical visit, the first dementia specialist visit and diagnosis were collected. Furthermore, details of the examinations (including imaging, blood and cerebrospinal fluid tests, and neuropsychological tests) and the dates of those tests until the diagnosis of AD were also collected and assessed.

Items related to the background of the patients included:

- Date of birth
- Sex

Items related to symptom onset and the first medical care provider the patient visited included:

- Presence of established medical care provider
- Individual who initially noticed symptom onset (patient, family member, friend, colleague in the workplace, medical worker, or other)
- Initial symptoms (i.e., symptoms that were present during the first 3 years after onset of AD)
- Type of medical care provider (hospital, clinic) and department of the first medical care provider that the patient visited (neurology, internal medicine, psychiatry, gerontology, neurosurgery, other)
- Date of first visit (year, month, day)
- Whether the patient was accompanied by another person during the first visit
- Marital status at symptom onset
- Household status at symptom onset (living together, living alone)
- Employment status at symptom onset (employed, not employed)
- Occupation at symptom onset
- Presence of on-treatment comorbidities at symptom onset (presence/absence of comorbidities, number of comorbidities, name of comorbidities, and duration of comorbidities)

Items related to the dementia specialist included:

- Date of first visit to the dementia specialist
- Transportation type used to visit the dementia specialist
- Travel time to the dementia specialist
- Family history of AD (biological parents, grandparents, and siblings)
- Time of symptom onset (year, month, day)
- Age at symptom onset
- Date of AD diagnosis (year, month, day)
- Educational level
- Number of visits to medical care providers from symptom onset to diagnosis (including visits not related to AD symptoms)
- Neuropsychological tests for dementia before AD diagnosis and date of each test (year, month, day)
- Imaging tests (computed tomography, magnetic resonance imaging, single photon emission computed tomography, amyloid positron emission tomography scan, other)
- Blood and cerebrospinal fluid biomarker testing (Aβ42, Aβ40, Aβ42/40, pTau181, tTau, NfL, other)

**Supplemental Figure 1.** First symptoms of Alzheimer’s disease (A, B) and most prominent initial symptoms during the first 3 years of symptom onset (C, D) in patients with EOAD vs LOAD.

EOAD: early-onset Alzheimer’s disease; LOAD: late-onset Alzheimer’s disease.

**Supplemental Figure 2.** Percentage of dementia specialist tests performed. (A) Imaging, (B) Blood and CSF biomarker testing, (C) Neuropsychological testing.

Blood tests were not performed in any of the cases.

*p ≤ 0.05, **p ≤ 0.01, ***p ≤ 0.001 (Fisher’s exact test).

ADAS-cog: Alzheimer’s Disease Assessment Scale-Cognitive Subscale; CDR: Clinical Dementia Rating; CSF: cerebrospinal fluid; CT: computed tomography; EOAD: early-onset Alzheimer’s disease; FAB: Frontal Assessment Battery; GDS: Geriatric Depression Scale; HDS-R: Hasegawa Dementia Scale-revised; LOAD: late-onset Alzheimer’s disease; MoCA-J: Montreal Cognitive Assessment-Japanese version; MMSE: Mini-Mental State Examination; MRI: magnetic resonance imaging; PET: positron emission tomography; SDS: Self-rating Depression Scale; SPECT: single photon emission computed tomography; STM-COMET: St. Marianna University School of Medicine’s Computerized Memory Test; TMT: Trail Making Test; WMS-R, LM: Wechsler Memory Scale-revised, logical memory.

**Supplemental Figure 3.** Time from symptom onset to AD diagnosis at Facility A, Facility B, Facility C, and Facility D.

AD, Alzheimer’s disease; EOAD, early-onset Alzheimer’s disease; LOAD, late-onset Alzheimer’s disease.

**Supplemental Table 1.** Factors examined in the multiple regression analysis.

| **For the time from symptom onset to AD diagnosis** |  |
| --- | --- |
| Diagnosis | EOAD |
|  | LOAD |
| Facility | A |
|  | B |
|  | C |
|  | D |
| Initial AD symptoms* | Aphasia |
|  | Visuospatial agnosia |
| Department of the first medical care provider that the patient visited | Neurology |
|  | Internal medicine |
|  | Psychiatry |
|  | Neurosurgery |
|  | Other + unknown |
| Whether the patient was accompanied by another person during the initial visit to the first medical care provider that the patient visited | Yes |
|  | None + unknown |
| Imaging test | MRI |
|  | SPECT |
|  | Amyloid PET scan |
| Positive cerebrospinal fluid test result | Aβ42 or Aβ42/40 ratio |
|  | pTau181 |
| Neuropsychological test | MMSE |
|  | HDS-R |
|  | WMS-R, LM |
|  | CDR |
|  | MoCA-J |
|  | ADAS-cog |
| **For the time from symptom onset to the first medical visit** |  |
| Diagnosis | EOAD |
|  | LOAD |
|  | A |
| Facility | B |
|  | C |
|  | D |
| Initial AD symptoms* | Visuospatial agnosia |
| Department of the first medical care provider that the patient visited | Neurology |
|  | Internal medicine |
|  | Psychiatry |
|  | Neurosurgery |
|  | Other + unknown |
| Whether the patient was accompanied by another person during the initial visit to the first medical care provider that the patient visited | Yes |
|  | None + unknown |
| Imaging test | MRI |
|  | SPECT |
|  | Amyloid PET scan |
| Positive cerebrospinal fluid test result | Aβ42 or Aβ42/40 ratio |
|  | pTau181 |
| Neuropsychological test | MMSE |
|  | HDS-R |
|  | WMS-R, LM |
|  | CDR |
|  | MoCA-J |
|  | ADAS-cog |
| **For the time from the first medical visit to the first dementia specialist visit** |  |
| Diagnosis | EOAD |
|  | LOAD |
| Individual who initially noticed symptom onset | Patient |
| Initial AD symptoms* | Cognitive impairment |
|  | Other |
| Department of the first medical care provider that the patient visited | Neurology |
|  | Internal medicine |
|  | Psychiatry |
|  | Neurosurgery |
|  | Other + unknown |
| Household status at symptom onset | Living together |
|  | Living alone or unknown |
| Educational level |  |
| Imaging test | MRI |
|  | SPECT |
|  | Amyloid PET scan |
| Positive cerebrospinal fluid test result | Aβ42 or Aβ42/40 ratio |
|  | pTau181 |
| Neuropsychological test | MMSE |
|  | HDS-R |
|  | WMS-R, LM |
|  | CDR |
|  | MoCA-J |
|  | ADAS-cog |
| **For the time from the first dementia specialist visit to AD diagnosis** |  |
| Diagnosis | EOAD |
|  | LOAD |
|  | A |
| Facility | B |
|  | C |
|  | D |
| Individual who initially noticed symptom onset | Colleague in the workplace |
| Initial AD symptoms* | Aphasia |
|  | Anxiety/depression |
|  | Apraxia |
|  | Other |
| Department of the first medical care provider that the patient visited | Neurology |
|  | Internal medicine |
|  | Psychiatry |
|  | Neurosurgery |
|  | Other + unknown |
| Number of visits to medical care providers from symptom onset to diagnosis |  |
| Imaging test | MRI |
|  | SPECT |
|  | Amyloid PET scan |
| Positive cerebrospinal fluid test result | Aβ42 or Aβ42/40 ratio |
|  | pTau181 |
| Neuropsychological test | MMSE |
|  | HDS-R |
|  | WMS-R, LM |
|  | CDR |
|  | MoCA-J |
|  | ADAS-cog |

*Symptoms that were present during the first 3 years after AD onset.

AD: Alzheimer’s disease; ADAS-cog: Alzheimer’s Disease Assessment Scale-Cognitive Subscale; CDR: Clinical Dementia Rating; EOAD: early-onset Alzheimer’s disease; HDS-R: Hasegawa Dementia Scale-revised; LOAD: late-onset Alzheimer’s disease; MMSE: Mini-Mental State Examination; MoCA-J: Montreal Cognitive Assessment, Japanese version; MRI: magnetic resonance imaging; PET: position emission tomography; SPECT: single-photon emission computed tomography; WMS-R, LM: Wechsler Memory Scale-revised, logical memory.

**Supplemental Table 2.** Sensitivity analysis of the primary endpoint.^*^

|  |  | Overall | EOAD | LOAD | Difference in mean (95% CI)  (EOAD − LOAD) |
| --- | --- | --- | --- | --- | --- |
| Time from symptom onset to AD diagnosis (weeks) | N | 129 | 74 | 55 | 39.2 (13.0, 65.4)  *t* = 2.96^†^  p = 0.004^†^  Z = −2.89^‡^  p = 0.004^‡^ |
|  | Mean ± SD | 112.0 ± 76.5 | 128.7 ± 79.6 | 89.5 ± 66.5 |  |
|  | Median (range) | 101.9 (3.1–322.4) | 110.4 (12.4–322.4) | 79.6 (3.1–280.7) |  |
| Time from symptom onset to the first medical visit (weeks) | N | 113 | 71 | 42 | 35.9 (10.3, 61.6)  *t* = 2.78^†^  p = 0.006^†^  Z = −3.03^‡^  p = 0.003^‡^ |
|  | Mean ± SD | 75.3 ± 68.4 | 88.7 ± 70.2 | 52.7 ± 59.4 |  |
|  | Median (range) | 55.4 (0.0–290.9) | 73.7 (0.0–290.9) | 40.6 (0.0–269.4) |  |
| Time from the first medical visit to the first dementia specialist visit (weeks) | N | 113 | 71 | 42 | 0.6 (−16.6, 17.8)  *t* = 0.07^†^  p = 0.944^†^  Z = −0.68^‡^  p = 0.496^‡^ |
|  | Mean ± SD | 31.1 ± 44.5 | 31.3 ± 43.5 | 30.7 ± 46.5 |  |
|  | Median (range) | 7.1 (0.1–179.3) | 7.1 (0.3–169.9) | 6.5 (0.1–179.3) |  |
| Time from the first dementia specialist visit to AD diagnosis (weeks) | N | 129 | 74 | 55 | 1.2 (−1.9, 4.4)  *t* = 0.76^†^  p = 0.449^†^  Z = −0.31^‡^  p = 0.760^‡^ |
|  | Mean ± SD | 7.6 ± 8.9 | 8.1 ± 10.2 | 6.9 ± 7.0 |  |
|  | Median (range) | 5.0 (0.0–59.0) | 5.0 (0.0–59.0) | 4.9 (0.0–37.3) |  |

^*^Outliers (data exceeding 3 standard deviations of the mean) were excluded.

^†^Student’s *t*-test.

^‡^Wilcoxon rank sum test.

AD: Alzheimer’s disease; EOAD: early-onset Alzheimer’s disease; LOAD: late-onset Alzheimer’s disease.
